# Study protocol for BeWEL: The impact of a BodyWEight and physicaL activity intervention on adults at risk of developing colorectal adenomas

**DOI:** 10.1186/1471-2458-11-184

**Published:** 2011-03-25

**Authors:** Angela M Craigie, Stephen Caswell, Caron Paterson, Shaun Treweek, Jill JF Belch, Fergus Daly, Jackie Rodger, Joyce Thompson, Alison Kirk, Anne Ludbrook, Martine Stead, Jane Wardle, Robert JC Steele, Annie S Anderson

**Affiliations:** 1 Centre for Research into Cancer Prevention and Screening, University of Dundee, UK; 2 Quality, Safety & Informatics Research Group, University of Dundee and Tayside Clinical Trials Unit, UK; 3 Vascular & Inflammatory Diseases Research Unit, University of Dundee, UK; 4 Dental Health Services Research Unit, University of Dundee, UK; 5 Colorectal Surgery, Ninewells Hospital and Medical School Dundee NHS Tayside, UK; 6 Directorate of Public Health, Dundee NHS Tayside, UK; 7 Department of Sport, Culture and The Arts, University of Strathclyde, Glasgow, UK; 8 Health Economics Research Unit, Institute of Applied Health Sciences, University of Aberdeen, UK; 9 Institute for Social Marketing, University of Stirling and The Open University, UK; 10 Health Behaviour Research Centre, Department of Epidemiology and Public Health, University College London, UK; 11 Department of Surgery and Molecular Oncology, Ninewells Medical School, University of Dundee, UK

## Abstract

**Background:**

Colorectal cancer (CRC) is the third most commonly diagnosed cancer and the second highest cause of cancer death in the UK. Most cases occur in people over 50 years and CRC often co-exists with other lifestyle related disorders including obesity, type 2 diabetes mellitus (T2DM) and cardiovascular disease (CVD). These diseases share risk factors related to the metabolic syndrome including large body size, abnormal lipids and markers of insulin resistance indicating common aetiological pathways.

**Methods/Design:**

This 3 year study will be a two-arm, multicentre, randomised controlled trial comparing the BeWEL lifestyle (diet, physical activity and behaviour change) programme against usual care. The pre-trial development will take 6 months and participants will be recruited over a 12 month period and undertake the intervention and follow up for 12 months (total 24 months recruitment and intervention implementation) with a further 6 months for data collection, analysis and interpretation.

Four hundred and fifty two participants who have had a colorectal adenoma detected and removed (through the national colorectal screening programme) will provide 80% power to detect a weight loss of 7% over 12 months.

Primary outcomes are changes in body weight and waist circumference. Secondary outcomes will include cardiovascular risk factors, psycho-social measures and intervention costs.

**Discussion:**

The results from this study will enhance the evidence base for lifestyle change in patients at higher risk of chronic disease including obesity related cancers.

International Standard Randomised Controlled Trials No: ISRCTN53033856

## Background

Colorectal cancer (CRC) is a major public health problem. It is the third most commonly diagnosed cancer and the second cause of cancer death in the UK [1]. Most cases (95%) occur in people over 50 years and CRC often co-exists with other lifestyle related disorders including obesity, type 2 diabetes mellitus (T2DM) and cardiovascular disease (CVD) [2,3].

Consistent with preventive effects for CVD and T2DM [4], there is evidence that physical activity protects against CRC, and that high levels of body fat (BMI >23 kg/m2) or a large waist circumference are associated with increased risk [5]. There is also evidence that red and processed meat and high alcohol intake increase CRC risk [5]. Thus a number of modifiable risk factors can be identified and addressed with potential benefit to risk of CRC, and proven benefit on risk reduction of T2DM and CVD.

Both public health and individual approaches are needed to assist health behaviour change and this is particularly important for individuals who are at risk of developing obesity related co-morbidities. The NHS CRC screening programme is increasing the identification of adenomas and the current programme roll-out provides a timely opportunity to offer risk factor reduction advice to older adults from all social and ethnic backgrounds who are at high risk of developing CRC (and other diet related conditions). The Scottish CRC screening data show that adenomas are most common in adults from areas of high deprivation (46% of colonoscopies vs 29% in adults from areas of low deprivation). Indeed, screen-positive men from the poorest backgrounds have a 50% chance of having an adenoma detected at colonoscopy (personal communication from R.J.C. Steele) and that is despite poorer uptake of screening in these areas.

Diabetes prevention trials have shown that lifestyle interventions that achieve a weight loss of 7% of initial body weight and at least 150 min/week of moderate intensity activity in adults with a BMI >25 kg/m2 reduce the incidence of T2DM [6-8] and have favourable effects on CVD risk factors [9]. It is likely that these lifestyle changes would also influence obesity-related neoplasia. Jacobs et al [10] identified obesity as a risk factor for short-interval (follow up 3 years) development of colorectal adenomas, particularly in men. Although it is unclear at what stage obesity impacts on adenoma development, there is evidence that adenoma risk increases among adults who have gained weight in the 5 years prior to colonoscopy [11]. Recent work from Japan which followed up 1,650 adults reported that the incidence of adenoma in those who had lost weight in the one year follow up period was significantly lower than in those who had maintained or gained weight [12]. The case for exploring cost-effective weight reduction strategies is also supported by long term follow-up trials of obesity surgery showing significant reduction in cancer mortality [13]. In addition, recent evidence has demonstrated that increasing physical activity in men aged over 50 shows a graded reduction in total mortality risk [14].

The major government strategy aimed at decreasing CRC burden is focused on early detection of the disease and national CRC screening programmes. CRC screening can also detect adenomas, which are the identifiable precursor lesions of CRC which can be removed by endoscopic procedures. This reduces the risk of subsequent cancer but the underlying (modifiable) risk factors which influence the development of new adenomas remain [15]. Current evidence suggests that the risk of new adenomas is around 40% after 3 years, although this may be higher in the morbidly obese [16]. Clinical encounters with healthy individuals who have had adenomas removed present an opportunity for risk factor reduction.

## Study Objectives

### Primary Outcomes

The main aim of the study is to evaluate the impact of a lifestyle (diet, physical activity and behaviour change) intervention programme ("BeWEL") on body weight change and waist circumference in healthy individuals attending routine NHS clinics who have had pre-cancerous bowel polyps removed.

### Secondary outcomes

We will examine whether there is a relationship between response to the BeWEL intervention and the participant's classification on the Scottish Index of Multiple Deprivation (SIMD) [17]. Cardiovascular risk factors will also be examined for changes in blood lipids, homeostasis model assessment (HOMA), as a measure of insulin resistance, blood pressure and Glycated haemoglobin (HbA1c). Lifestyle habits, self-assessed health, self-efficacy, perceived acceptability of the programme, and intervention costs will also be assessed.

## Methods/Design

Ethical approval for the study has been received from NHS Tayside Regional Ethics Committee (see later). All participants will receive a written participant information sheet explaining the trial and all will be asked to give written consent.

### Study/trial design

The study will be a two-arm, multicentre, randomised controlled trial comparing the BeWEL intervention with usual care and will last for 3 years.

### Treatment period and follow up

All baseline measures will be made prior to group allocation, and follow up measures (at 3 and 12 months) will be subject to a strict protocol with researchers blind to group allocation. Intervention group participants will have face-to-face intervention contact on 3 occasions during the first 3 months followed by bi-monthly (telephone/email) contact until 12 months. Participants in both groups will be asked to complete a single page exit questionnaire on the acceptability of the study procedures.

Measures of CVD risk (lipids, HOMA and blood pressure) have been included as part of good clinical practice (given that many of these individuals will have early signs of metabolic syndrome or CVD) as a check for unintended consequences. Secondary outcomes are not part of our sample size calculations (which is normal practice) but we believe they will add value without excessive participant burden and will be available for future meta-analysis/review purposes and hypothesis generating in this area.

### Process Evaluation

Programme acceptability will be explored post-intervention with in-depth exit interviews with a random sample of 30 intervention participants. Interviews will cover participants' initial expectations and motivations regarding the programme, the extent to which these were met or not by their subsequent experiences, and factors influencing their ability to make the recommended lifestyle changes.

### Measures/assessment instruments

Measures at all outcome points will be completed face-to-face. Details of the outcomes that are collected at the different time points are detailed in Table 1 below.

**Table 1:** Outcome Measures (B = baseline; 3F = 3 month follow-up, 12F = 12 month follow-up)

	Measure	When
Primary Outcome		

BMI (weight & height)	Calibrated scales & stadiometer	B, 3F, 12F

Waist circumference	Tape measure	B, 3F, 12F

Lipid profile	Blood test	B, 3F, 12F

Blood pressure	Sphygmonanometer	B, 3F, 12F

HOMA	Blood test	B, 3F, 12F

HbA1c Blood test B, 3F, 12F	Blood test	B, 3F, 12F

Secondary Outcomes		

Diet	DINE diet questionnaire	B, 3F, 12F

Physical activity	Sensewear physical activity monitor (7 days)	B, 3F, 12F

General health and self efficacy	Questionnaire	B, 3F, 12F

Programme acceptability	In-depth interview with clinic and counselling staff	Post intervention

### Centre/practice and/or participant selection

The intervention study will be delivered in 3 centres (Tayside, Forth Valley and Ayrshire and Arran) and the lead colorectal cancer clinicians at each site have established that the colorectal/endoscopy teams are willing to participate in this trial.

### Inclusion criteria

Screening adenoma patients aged 50 to 74 years with a BMI >25 Kg/m^2 ^who are physically able to undertake exercise requirements and are able to provide informed consent.

### Exclusion criteria

Patients will not be eligible for the trial if they have a normal colonoscopy, are insulin-dependent patients with diabetes or are diagnosed with cancer.

### Participant Recruitment

All patients who undergo screening colonoscopy procedures and are found to have benign adenomas are sent a letter reassuring them of their findings and re-enforcing the importance of follow up surveillance (further colonoscopy). The first contact about the study will be made at this point. This will comprise a second (brief) letter from the colorectal cancer surgeon, enclosed with the screening results, endorsing the study and encouraging them to read an information sheet which will be sent by the study team within two weeks. The participant information sheet will be sent by the research nurse along with a covering letter of invitation, a reply slip and a pre-paid reply envelope. Those who state their interest in taking part will be screened for eligibility over the telephone, and (if eligible) invited to the study centre (with a spouse or other family support member) to provide informed consent and undergo baseline measures. The trial manager will be responsible for ensuring that allocation at each site follows standard procedures with even distribution into each condition.

All participants will be provided with written lifestyle advice (British Heart Foundation leaflet: So you want to lose weight for good: A guide to losing weight for men and women) so that it is less obvious to participants which arm of the trial they have been allocated to. Staff responsible for recruitment and follow-up will not deliver the intervention. The intervention will be delivered by trained lifestyle counsellors. All baseline measures will be made prior to group allocation and follow up measures will be subject to a strict protocol with researchers blind to group allocation.

### Informed consent

Those who state their interest in taking part will be given any further information they require and, if eligible, invited to the study centre (with a spouse or other family support member) to provide informed consent and undergo baseline measures.

### Registration

A record of individuals who were invited to participate in the trial, whether consent to be contacted by telephone was given or declined, and their eligibility will be kept by the research nurse who initially contacts potential participants. The research nurse will also keep a log of any individuals who declined at the trial consent meeting. A case report form (CRF) will be completed for all consented individuals. Details of a nominated contact (e.g. spouse or friend) will also be collected to facilitate participant follow-up. The research nurse will use the trial data management system to enter and store data on all eligible individuals. Recruitment information will also be monitored at regular intervals by comparing this to the number being approached and numbers declining.

### Non-registration

Individuals can decline to consent to taking part in the study and their medical care will not be affected.

### Withdrawal and loss to follow-up

Individuals have the right to withdraw consent for participation in any aspect of this trial at any time. Care from other services will not be affected at any time by declining to participate or withdrawing from the trial.

We will make every effort to reduce loss to follow-up. We will visit participants at home should they wish, which we expect to improve response rates and loss to follow-up. If a participant misses one follow-up we will try to arrange with them on two further occasions. We will reimburse any travel costs.

## Trial intervention

### The Intervention Group (IG)

Intervention participants will receive the "BeWEL" personalised intervention programme, personal (body weight) scales, and invitations to undertake supervised monthly body weight. The BeWEL personalised, multiple contact, intervention programme will be largely based on the US diabetes prevention programme (http://www.bsc.gwu.edu/dpp/index.html), which includes (a) goal-setting for weight, activity, and calorie intake; (b) self-monitoring to achieve these goals; (c) frequent contact to provide accountability and sustain focus; (d) use of problem-solving and other "toolbox" strategies to address goals and potential barriers to achieving them; and (e) emphasis on managing individual high-risk situations. The approach will take particular care to emphasise the importance of regular self-weighing which is widely associated with greater weight loss and weight prevention (showing a 1 to 3 BMI unit advantage over individuals who do not self-weigh frequently) [18,19].

### The Usual Care (comparison group)

Usual care participants will be given a general leaflet on healthy lifestyle that is widely available in the NHS setting. This will ensure that all participants receive some lifestyle advice which at the moment is given out on an ad hoc basis. Whilst not a 'no treatment' control group, 'no treatment' is not current practice; individuals may receive lifestyle information. We will collect weight information in the comparison group, which is not a routine measurement for this group of patients. Although we recognise that this may influence behaviour in the comparison group, we anticipate that the effect will be small and it is essential for collecting data for the primary outcome.

**Figure 1:**
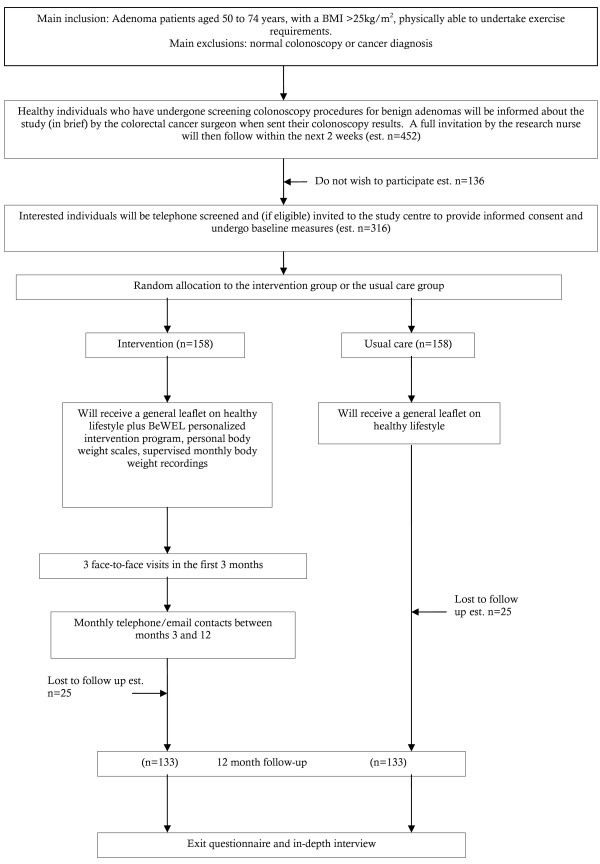
**Participant flow diagram**.

Participants in both groups will be reminded of their follow-up appointments by advance telephone call. Figure 1 below illustrates the participant pathway through the trial.

### Serious adverse events

No serious adverse events (SAE) are anticipated. However if any SAE occur, this will be drawn to the immediate attention of the participant's General Practitioner.

## Statistical considerations

### Randomisation

Once individuals have consented to the study and had baseline measurements taken they will then be randomly allocated by a permuted-block technique, with block sizes of four or eight and with stratification by trial site into the usual care group or an intervention group. Randomisation will be done by email (agreed protocol in place to ensure this procedure can be undertaken during all office hours) to the trial centre.

### Sample size

To demonstrate a 7% weight loss with 80% power it is estimated that 133 participants would be required to complete each arm of the study. The sample size is based on the aim of achieving 7% weight loss at 12 months which has been demonstrated to be clinically effective in diabetes prevention. In the Bowel Health to Better Health (BHBH) study [20] the mean body weight was 85.4 (± 17.3) kg. Allowing for a drop out rate of 16%, this would mean enrolment of 158 into each arm of the study. With a 70% recruitment rate this would require 226 eligible subjects in each arm. It is estimated that 81% of clinic subjects would meet the eligibility criteria thus a pool of 558 subjects are required from which to recruit for the study. Current figures suggest that 200 patients are diagnosed with adenoma each year at each of the 3 participating sites, thus a one year recruitment period should allow for any variation in the estimated figures and easily permit recruitment to the intended design.

## Analysis

### Main analysis

Randomisation will be stratified by site. The primary analyses will be carried out under intention-to-treat but secondary analyses will explore the effect of treatment received. The main analyses will involve standard two-sample comparisons (parametric or nonparametric as dictated by the distribution of the data) looking at effect sizes at 3 and 12 months. As these are mainly continuous outcomes, this will involve t-tests or Mann-Whitney tests as well as repeated measures. All analyses will be stratified by centre. Differences by site will be explored and, if appropriate, the site can be entered in a mixed model as a random effect. The balance of characteristics between treatment and control arms will be tabulated and if differences are noted, adjustment will be made for these in linear regression models. Pre-specified subgroup analyses for socioeconomic status will be carried out by including the appropriate treatment interaction term. Thus sub-group analyses will be exploratory and hypothesis generating rather than hypothesis testing.

### Qualitative analysis

A thematic analysis of interview transcripts will be undertaken, exploring such themes as what factors influence decisions to engage in the programme, how uptake is influenced by socio-economic status, the practical barriers and opportunities for facilitating physical activities and changes in dietary habits, and the perceived acceptability of the programme to participants and families.

### Economic analysis

The economic analysis will take the form of a cost-consequence analysis (CCA), in which all potential costs and benefits will be identified, measured and valued where appropriate (differences expected between groups) and feasible. The analysis will be undertaken from both an NHS and societal perspective. For interventions that require individual behaviour change to be successful, it is particularly important to consider the costs and benefits from the perspective of individual participants and their families [21]. These will be explored in the context of the CCA and in more depth in the post-intervention interviews. CCA also allows for a flexible presentation of results in contexts where there is more than outcome of interest.

The NHS costs of the intervention will be assessed according to the intervention protocol, with sampling to assess length and frequency of contacts, fidelity of delivery and staff time. The analysis will also be informed by the qualitative part of the research. NHS benefits related to reduced service use as a consequence of healthier lifestyles will be modelled from secondary data and relevant literature. This modelling will allow estimates to be made of the potential longer term benefits of behaviour change.

## Data storage & retention

Data management will be handled by the Tayside Clinical Trials Unit with data being held according to GCP requirements. As per MRC requirements, data will be held for a minimum of ten years from completion of the project.

## Ethical approval, research governance and data access

Ethical approval was obtained from the Tayside Research Ethics Committee via IRAS, (Tayside Committee on Medical Research Ethics B. Ref No. 10/S1402/34, Approval granted 23rd July 2010) with other participating centres providing site-specific approval as per normal IRAS procedures. NHS Research and Development (R&D) approval was obtained from all participating NHS Boards prior to the start of the trial (Tayside R&D Project ID 2010ON16), (Ayrshire and Arran R&D Project ID 2010AA047) and (Forth Valley R&D Reference FV539). Further advice and support on governance and good clinical practice (GCP) issues will be provided by the Tayside Clinical Trials Unit. BeWEL will make use of the University of Dundee's Standard Operating Procedures for tasks such as obtaining consent, managing and archiving data, access to trial data, training and how to handle breaches of GCP. The trial has been submitted to The International Standard Randomised Controlled Trials (ISRCT) and allocated the number ISRCTN53033856.

## Study/trial sponsorship

The University of Dundee is the sponsor of this trial.

## Discussion/Rationale for current study

Behaviour change programmes that target high risk groups can be more effective at the individual level than those targeting the population at large [22]. It is possible that adults who have had a 'health scare' (e.g. diagnosis of adenoma) experience a 'teachable moment' [23] in which they will be more motivated to engage in and adhere to lifestyle advice. Current evidence does not suggest that adenoma diagnosis per se is associated with a change in health behaviours [24]. This client group has been reported to have low levels of nutrition knowledge and little awareness of the link between diet and cancer [25]. However, there is evidence that they would welcome advice on healthy eating, with face-to-face mode being preferred [26]. We have also demonstrated that diet and activity in this group is inconsistent with current guidelines [27].

The NHS setting provides i) an existing framework for obesity intervention work particularly when co-morbidities (including colorectal adenomas) are present, ii) access to clinicians who can endorse behaviour change in healthy volunteers who have had a health scare and are potentially motivated towards prevention action, and iii) the potential for long term follow-up. The advantage of a hospital setting is that it provides a central location that all participants will have visited and therefore know the route and location. This is important for men who may rarely have visited their primary care practice. In addition, the hospital base re-enforces clinical endorsement and the importance of the desired outcome. The results from the recent TIME2ACT [27] study of increasing physical activity in patients with diabetes suggests that response is higher when interventions are delivered in a hospital setting because of the perceived medical endorsement and the sense that the intervention is an integrated part of care. From a practical perspective it increases the likelihood of this type of intervention being feasible (e.g. it would be difficult to follow up individual patients from a large geographical area who could be followed up in the community), and there are existing NHS obesity services in the hospital outpatient setting.

The results from this study will assess the impact of the BeWEL intervention on body weight and CVD risk. The study will also provide a platform (feasibility evidence) for the long-term evaluation of BeWEL on adenoma development in high risk, healthy participants and demonstrate the potential for lifestyle interventions to be initiated in routine NHS clinics. Furthermore the work will increase understanding of participant engagement, barriers, opportunities and experiences of lifestyle management programmes, and examine the cost-effectiveness of the intervention procedures in this NHS setting.

The results of the study have implications for the prevention and delay of onset of all obesity related chronic diseases that are major causes of morbidity and death in the UK. The study is of direct relevance to the NHS and has the potential to significantly enhance current government action on the prevention of a range of obesity-related disorders. The results will also have implications for other studies that aim to transfer research findings into routine care. The process study data (participant experience) will be relevant to research beyond obesity management.

Beyond the end of the project, 5-year follow-up measures of adenoma occurrence will be assessed from routine NHS screening data collection. Thus, although not powered to detect disease end-points, the BeWEL data will provide indicative data (feasibility data on ability to achieve weight loss) of a lifestyle trial for chronic disease outcomes.

## Competing interests

The authors declare that they have no competing interests.

## Authors' contributions

All authors contributed to the design and writing of the protocol. All authors have approved the final manuscript.

## Pre-publication history

The pre-publication history for this paper can be accessed here:

http://www.biomedcentral.com/1471-2458/11/184/prepub
